# Early postnatal maternal trait anxiety is associated with the behavioural outcomes of children born preterm <33 weeks

**DOI:** 10.1016/j.jpsychires.2020.09.010

**Published:** 2020-12

**Authors:** I. Kleine, S. Falconer, S. Roth, S.J. Counsell, M. Redshaw, N. Kennea, A.D. Edwards, C. Nosarti

**Affiliations:** a Centre for the Developing Brain, Faculty of Life Sciences & Medicine, King's College London, St Thomas' Hospital, London, SE1 7EH, UK; b Policy Research Unit in Maternal Health and Care, National Perinatal Epidemiology Unit, Nuffield Department of Population Health, University of Oxford, Oxford, UK; c St George's Hospital NHS Trust, Blackshaw Road, London, SW17 0QT, UK; d Department of Child and Adolescent Psychiatry, Institute of Psychiatry, Psychology and Neuroscience, King's College London, London, SE5 8AF, UK

**Keywords:** Neonatology, Preterm, Perinatal, Anxiety, Child behaviour

## Abstract

Maternal ante- and postnatal anxiety have been associated with children's socio-emotional development. Moreover, maternal anxiety has been studied as both a contributing factor and consequence of preterm birth, and children born preterm are more likely to develop behavioural problems compared to term-born controls. This study investigated the association between maternal anxiety measured soon after birth and mental health in 215 ex-preterm children, born at <33 weeks, who participated in the Evaluation of Preterm Imaging Study. Children were followed-up at a median age of 4.6 years (range 4.2–6.6), and received behavioural and cognitive evaluation. Maternal trait anxiety was assessed with the Spielberger State-Trait Anxiety Index at term corrected age. Primary outcome measures were children's Strengths and Difficulties Questionnaire (SDQ) and Social Responsiveness Scale 2 (SRS-2) scores, indicative of generalised psychopathology and autism symptomatology, respectively. IQ was assessed with the Wechsler Preschool and Primary Scales of Intelligence. The final sample, after excluding participants with missing data and multiple pregnancy (*n* = 75), consisted of 140 children (51.4% male). Results showed that increased maternal trait anxiety at term corrected age was associated with children's higher SDQ scores (β = 0.25, 95% CI 0.09–0.41, *p* = 0.003, f^2^ = 0.08) and SRS-2 scores (β = 0.15, 95% CI 0.02–0.28, *p* = 0.03, f^2^ = 0.04). Our findings indicate that children born preterm whose mothers are more anxious in the early postnatal period may show poorer mental health outcomes at pre-school age. Further research is needed to investigate preventative measures that can be offered to high-risk premature babies and their families.

## Highlights

• Postnatal maternal anxiety may predict behavioural problems in ex-preterm children.
• Postnatal maternal anxiety is not associated with IQ in ex-preterm children.
• Further research needed in the prevention of mental illness in ex-preterm children.

## 1 Introduction

The foetal and early postnatal time encompasses a critical period of development, during which alterations to typical maturational patterns have been associated with neurodevelopmental sequelae (Meredith, 2015). Preterm birth, affecting over 7% of babies in the UK (National Institute for Health and Care Excellence, 2015), can disrupt typical neurodevelopment (Ment and Vohr, 2008) and has significant consequences for later cognitive, behavioural and psychiatric outcomes (Anderson, 2014; Johnson and Wolke, 2013; Nosarti et al., 2012). The role of parental stress in defining offspring's behavioural outcomes is of increasing interest to public health, as over 10% of new mothers (Dennis et al., 2017), as well as fathers (Leach et al., 2016), suffer from postnatal anxiety, and is of particular relevance to preterm infants, whose parents are at increased risk of distress (Carson et al., 2015), and who themselves are already at heightened risk of long-term neurodevelopmental difficulties.

Recent studies have investigated the role of both ante- and postnatal parental stress and anxiety in child development and behaviour (Bendiksen et al., 2020; Kvalevaag et al., 2013; Polte et al., 2019; Rees et al., 2019), although inconsistencies between study methodologies and findings make definite conclusions difficult (Rees et al., 2019; Tarabulsy et al., 2014). Postnatal maternal stress and anxiety are believed to have significant long-term implications for child brain maturation (Lautarescu et al., 2020; Qiu et al., 2013) and behavioural development (Field, 2018), particularly in very preterm infants (Hadfield et al., 2017). Possible mechanisms mediating these associations include reduced maternal sensitivity (Kertz et al., 2008; Nicol-Harper et al., 2007), unpredictability of maternal behaviour (Davis et al., 2017), and altered mother-infant attachment (Hadfield et al., 2017; Stevenson-Hinde et al., 2013). However, several factors are likely to interact to influence children's developmental outcome, and therefore it is difficult to disentangle the multi-factorial developmental origins of childhood psychiatric disorders.

Maternal anxiety is of particular relevance to the study of prematurity, as it can be both a contributing factor to (Doktorchik et al., 2018; Dole et al., 2003; Staneva et al., 2015) and consequence of preterm birth (Carson et al., 2015; Edwards et al., 2018; Henderson et al., 2016).

In light of the elevated risk of behavioural disorders, including attention deficit hyperactivity disorder (ADHD) and autism spectrum disorder (ASD), faced by preterm infants (Johnson and Wolke, 2013), it is important to understand the role of potentially modifiable factors in neonatal care, in order to guide the development of targeted interventions aimed at strengthening children's mental health.

Past studies have investigated the role of later parental anxiety in determining offspring outcomes in population samples and retrospective cohorts, but, to our knowledge, none have examined the effect of early postnatal anxiety in very preterm infants. Thus, we aimed to investigate the developmental impact of postnatal maternal anxiety in a multi-centre longitudinal cohort of high-risk preterm infants followed up into childhood, incorporating clinical and socio-demographic factors, to further our understanding of the aetiology of the neurodevelopmental and behavioural problems associated with very preterm birth. We hypothesised that early postnatal maternal anxiety would be negatively associated with very preterm children's long-term mental health outcomes.

## 2 Materials and methods

### 2.1 Sample

Study participants were enrolled into the Evaluation of Preterm Imaging study (e-Prime, NCT01049594; EudraCT 2009-011602-42). 511 infants were recruited at birth, between April 2010 and July 2013, from hospitals in the North and South-West London Perinatal Network. Inclusion criteria were: birth <33 weeks’ gestation, mother aged over 16 years and not a hospital inpatient. Exclusion criteria were: major congenital malformation, previous MRI, metallic implants, parents unable to speak English, or family subject to child protection proceedings. The recruitment process and MRI protocol for the e-Prime study have been outlined previously (Edwards et al., 2018).

**Fig. 1:**
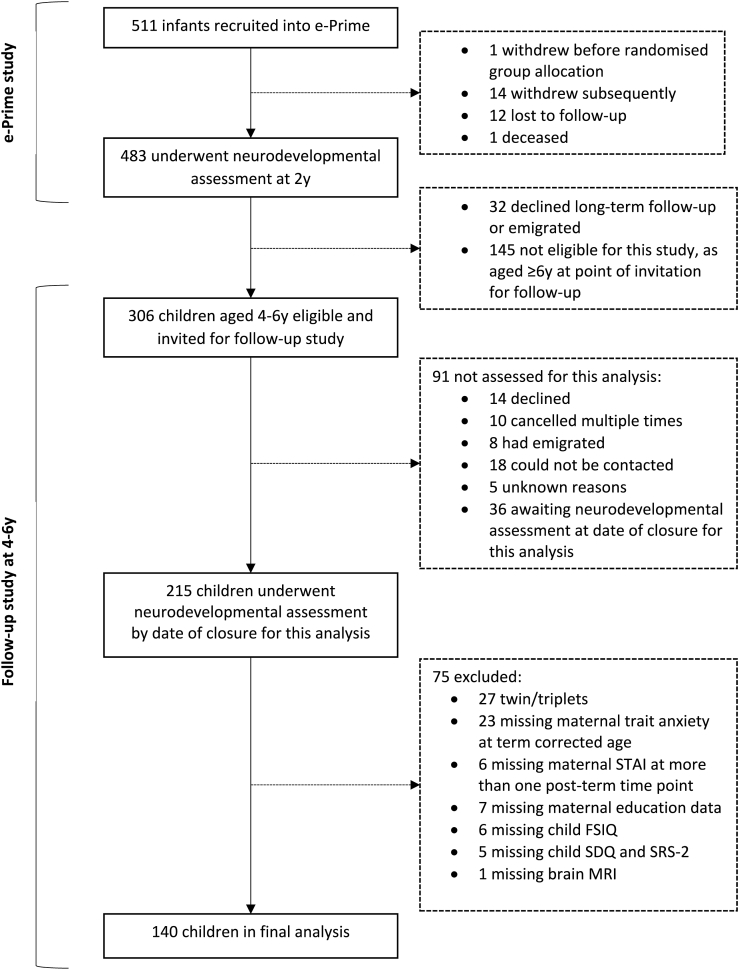
Recruitment flow chart.

This follow-up study of the e-Prime cohort invited children aged 4–6 years to the Centre for the Developing Brain, St Thomas' Hospital, London, for neurodevelopmental assessment, as outlined in Fig. 1. Invitations for follow-up were sent in chronological order of birth to all 306 children who were eligible based on age; 55/306 (18.0%) did not participate, and 36/306 (11.8%) were awaiting neurodevelopmental assessment at date of closure for this analysis (February 22, 2019). Thus, a convenience sample of 215/306 children (70.3%), created by families that were eligible for and willing to undergo testing at 4-6 years, was assessed. Written informed consent was given by the children's carer(s), following protocols that were approved by the Stanmore Research Ethics Committee (14/LO/0677). This study was conducted in accordance with the World Medical Association's Code of Ethics (Declaration of Helsinki).

### 2.2 Maternal variables

Maternal age, education, marital status and postcode were collected at enrolment into the e-Prime study. Index of Multiple Deprivation (IMD) score was computed from the current maternal postcode (Department for Communities and Local Government, 2011; https://tools.npeu.ox.ac.uk/imd/) and provided a proxy for family socio-economic status.

*Maternal trait anxiety* was evaluated using the State-Trait Anxiety Inventory (STAI) (Spielberger et al., 1983) when children were first assessed at term-equivalent age, then at 14 days, 12 months and 22 months age-corrected (Edwards et al., 2018). Missing values in the STAI-trait were imputed if ≤ 10% of questions were unanswered, by averaging responses for the answered questions (*n* = 13/140 at term-equivalent age, *n* = 2/140 at 14 days, *n* = 7/140 at 12 months, and *n* = 6/140 at 22 months corrected age). State anxiety refers to a transitory emotional state, probing how participants feel “right now”, and fluctuates over time (Spielberger et al., 1983); in the ePrime sample we previously reported a lower maternal STAI state score after infants received MRI imaging (Edwards et al., 2018). Trait anxiety, on the other hand, refers to how participants feel “in general” and indicates a person's proneness to responding anxiously to stressful situations; it is thought to be stable over time (Spielberger et al., 1983), although its stability has been questioned in perinatal cohorts (Meades and Ayers, 2011). In this study, we used maternal trait anxiety measures taken at the child's term-equivalent age, the most proximal timepoint to preterm birth, to provide an early postnatal estimation of a mother's tendency to respond anxiously to potentially stressful events. A measure of continuous postnatal maternal anxiety was computed using the mean maternal trait scores from 14 day, 12 month and 22 month age-corrected assessments. There was a significant difference between the maternal trait anxiety score assessed at child's term-equivalent age (M = 37.2) and the mean postnatal score (M = 35.8) (t (139) = 2.42, *p* = 0.02).

### 2.3 Child variables

Infant clinical and socio-demographic characteristics were obtained at enrolment into the e-Prime study and included: sex, gestational age at birth, birth weight, mode of delivery, antenatal steroids, singleton/multiple birth, days ventilated, use of surfactant, days given total parenteral nutrition (TPN), diagnosis of necrotising enterocolitis (NEC), receipt of expressed breast milk (EBM) in the Neonatal Intensive Care Unit (NICU), and brain MRI findings. Qualitative MRI classification was performed by SJC for the 215 infants assessed at 4–6 years, and included: major lesions defined as cystic periventricular leukomalacia, >10 punctate white matter lesions, and/or grade 3 or 4 germinal matrix haemorrhage; minor lesions defined as any other lesions; and no lesions.

### 2.4 Outcome variables

Behavioural and cognitive assessments were conducted when children had a median corrected age of 4.6 years (range 4.2–6.6). *Behavioural outcomes* were assessed with the parent-completed Strengths and Difficulties Questionnaire (SDQ) (Goodman, 2001) and Social Responsiveness Scale 2 (SRS-2) (Constantino and Gruber, 2012). The SDQ is commonly used as a screening tool for general childhood psychopathology. It comprises 25 items assessing psychological attributes, categorised into five subsets: emotional symptoms, conduct problems, hyperactivity/inattention, peer relationship problems and prosocial behaviour. The first four subsets are combined into a total difficulties score, which was used as our SDQ outcome measure. Higher scores indicate more behavioural problems and are categorised into four bands: average (0–13), slightly raised (14–16), high (17–19) and very high (20–40) (YouthinMind, 2016). The SRS-2 is a screening and assessment tool for autism spectrum disorder, providing subscale scores for social awareness, social cognition, social communication, social motivation, restricted interests and repetitive behaviours, as well as an overall total score; gender-normed T-scores are also given. The validity of the US-normed questionnaire has been supported in a British population sample (Wigham et al., 2012). This study used the total SRS-2 T-score as the outcome measure. Higher scores indicate greater deficits in social behaviour and are categorised into four groups: within normal limits (0–59), mild deficit (60–65), moderate deficit (66–75) and severe deficit (≥76) (Constantino and Gruber, 2012).

*Cognitive outcomes* were assessed with the Wechsler Preschool and Primary Scales of Intelligence IV (WPPSI-IV); 5 subscales – verbal comprehension, visual spatial, fluid reasoning, working memory and processing speed – are totalled to provide a full-scale IQ score (FSIQ) (Wechsler, 2012).

### 2.5 Analyses

215 children were followed up longitudinally. Multiple pregnancies were identified in the cohort; where more than one twin/triplet was included in the sample, the Excel RAND function was used to randomly select only one from each pair/triplet for analysis (excluded *n* = 27). The following children were also excluded: missing maternal education data (*n* = 7); missing maternal STAI at term corrected age (*n* = 23); missing maternal STAI at more than one post-term time point (*n* = 6); missing neonatal brain MRI (*n* = 1); missing child's IQ (*n* = 6); and missing both SDQ and SRS-2 scores (*n* = 5). Of the remaining 140 cases, 1 child did not have an SDQ score, and 5 children did not have SRS-2 scores. Those analysed in this study (*n* = 140) did not differ from those excluded with incomplete data (*n* = 75) in terms of gestational age (t (213) = –0.34, *p* = 0.73) and birth weight (t (213) = 0.74, *p* = 0.46). Comparisons of baseline socio-demographic and clinical characteristics between our cohort (*n* = 140) and those who took part in the e-Prime study but were not followed up (*n* = 370) are shown in Table 1 and Table 2.

**Table 1:** Family socio-demographic characteristics.

	e-Prime sample (n=510)	Follow-up sample (n=140)	Not followed up (n=370)	p
Index of Multiple Deprivation quintiles, n (%)				
1 (least deprived)	91 (17.8)	32 (22.9)	59 (15.9)	0.25
2	82 (16.0)	26 (18.6)	56 (15.1)	
3	129 (25.2)	32 (22.9)	97 (26.2)	
4	135 (26.4)	31 (22.1)	104 (28.1)	
5 (most deprived)	73 (14.3)	19 (13.6)	54 (14.6)	
**Maternal age at infant’s birth (years), mean [95% CI]**	32.9 [32.4 – 33.4]	34.3 [33.3 – 35.3]	32.4 [31.8 – 33.0]	**0.001**
**Maternal age upon leaving formal education, n (%)**				
≤16 years	49 (9.6)	9 (6.4)	40 (10.8)	**0.006**
17-19 years	76 (14.9)	23 (16.4)	53 (14.3)	
≥19 years	348 (68.1)	105 (75.0)	243 (65.7)	
Still in full-time education	16 (3.1)	3 (2.1)	13 (3.5)	
Not stated	21 (4.1)	0 (0)	21 (5.7)	

Characteristics given for this study’s follow-up sample (n=140), compared to those of the e-Prime sample not followed up (n=370).

**Table 2:** Infant demographic and clinical characteristics.

	e-Prime sample (n=510)	Follow-up sample (n=140)	Not followed up (n=370)	p
**Sex: Male, n (%)**	253 (49.5)	72 (51.4)	181 (48.9)	0.73
**Gestational age at birth (weeks), median [range]**	30 [23 – 32]	30 [23 – 32]	29 [23 – 32]	0.72
**Birthweight (g), median [range]**	1274 [552 – 2600]	1280 [600 – 2600]	1268 [552 – 2510]	0.38
**Mode of delivery, n (%)**				
Emergency caesarean	292 (57.1)	70 (50.0)	222 (60.0)	0.07
Elective caesarean	44 (8.6)	11 (7.9)	33 (8.9)	
Vaginal	170 (33.3)	59 (42.1)	111 (30.0)	
Unknown	4 (0.8)	0 (0)	4 (1.1)	
**Antenatal steroids course, n (%)**				
Full	431 (84.3)	112 (80.0)	319 (86.2)	0.27
Partial	64 (12.5)	22 (15.7)	42 (11.4)	
None	15 (2.9)	6 (4.3)	9 (2.4)	
**Multiple birth, n (%)**	164 (32.2)	19 (13.6)	145 (39.2)	**0.000**
**Days ventilated, median [range]**	1 [0 – 65]	0 [0 – 41]	1 [0 – 65]	0.48
**Surfactant, n (%)**				
Given	277 (54.3)	79 (56.4)	198 (53.5)	0.72
Not given	233 (45.7)	61 (43.6)	172 (46.5)	
**Days Total Parenteral Nutrition, median [range]**	6 [0 – 151]	6 [0 – 103]	6 [0 – 151]	0.40
**Necrotising Enterocolitis, n (%)**				
No	468 (91.8)	133 (95.0)	335 (90.5)	0.37
Yes – medical	35 (6.9)	5 (3.6)	30 (8.1)	
Yes – surgical	7 (1.4)	2 (1.4)	5 (1.4)	
**Expressed Breast Milk, n (%)**				
Given	438 (85.9)	118 (84.3)	320 (86.5)	0.68
Not given	72 (14.1)	22 (15.7)	50 (13.5)	

Characteristics given for this study’s follow-up sample (n=140), compared to those of the e-Prime sample not followed up (n=370). Median and range used for non-normal distributions.

The following variables were entered in our model: maternal trait anxiety scores at term corrected age; child's gestational age at birth; days ventilated; sex; brain MRI classification; child's FSIQ score; child's corrected age at assessment; IMD score; and maternal education, categorised as higher educational level (leaving full-time education aged ≥19 years) and lower education level (leaving full-time education aged ≤18). The same analyses were repeated using mean postnatal maternal trait anxiety scores, in order to distinguish between possible effects of early postnatal vs continuing postnatal anxiety. It was not possible to model early postnatal maternal anxiety together with continuing postnatal anxiety, due to multicollinearity (r = 0.78, eTable 1 in the Supplement).

The following variables were not included in our analysis: antenatal steroids, which only 6/140 neonates (4.3%) did not receive; NEC, which was diagnosed in only 7/140 (5.0%) of cases; receipt of EBM in NICU, as only 22/140 (15.7%) did not receive any EBM; days TPN, due to collinearity with days ventilated (r = 0.78) and gestational age (r = −0.63, eTable 1 in the Supplement); surfactant use, due to its association with fewer days ventilated (t (79.4) = 5.64, *p* = 0.000); and birthweight, due to its correlation with gestational age at birth (r = 0.80, eTable 1 in the Supplement).

Descriptive statistics, two-tailed Pearson correlations, part correlations, collinearity diagnostics, two-tailed t-tests, Chi-squared and Fisher's Exact tests were performed in IBM SPSS Statistics for Windows, Version 25.0 (IBM Corp., 2017). Model selection, regression and diagnostics were performed with RStudio (RStudio Team, 2016). To identify the key variables affecting total SDQ and SRS-2 scores at pre-school age, linearity between variables was confirmed and best-fit linear models were developed using an automated model selection process (Calcagno, 2019). Model comparison was performed using Akaike Information Criterion (AIC). Standardised coefficients were calculated by regressing Z-scored variables. Collinearity diagnostics confirmed Variance Inflation Factor <2 and Condition Index <2 for all SDQ and SRS-2 model variables. The normal Q-Q plot for the SDQ model with maternal anxiety at term corrected age appeared acceptable, and the fitted:residual plot suggested heteroscedasticity; Shapiro-Wilk test showed non-normality of residuals (W = 0.97, *p* = 0.002) and studentised Breusch-Pagan test showed evidence of heteroscedasticity (BP = 13.43, *p* = 0.02). Analysis of plots and diagnostic statistics for the SRS-2 model with maternal anxiety at term corrected age revealed marked right-skew (W = 0.92, *p* < 0.000) and heteroscedasticity (BP = 11.68, *p* = 0.02). However, in light of literature supporting the validity of linear regression even in cases of non-normality with sufficiently large sample size (Lumley et al., 2002), we used multiple linear regression. Robust standard errors were computed using a heteroscedasticity-consistent estimator, with the HC3 bias adjustment (Long and Ervin, 2000; Zeileis et al., 2019). Models of SDQ and SRS-2 using mean postnatal maternal trait anxiety had no evidence of multicollinearity, but showed non-normality and heteroscedasticity of residuals; robust standard errors were used. Outliers were screened for using Cook's distance, which was <0.5 in all models. Effect size was calculated using Cohen's f^2^ (Cohen, 1988), with part correlations for predictors that were significant at *p* < 0.05. Harman's single factor test was used to test for common method bias amongst the variables (Podsakoff et al., 2003); unrotated principal axis factoring showed that a single factor explained 12.9% of variance in the SDQ and SRS-2 models at term corrected age.

Regression analyses were also performed to determine the effect of maternal anxiety on children's cognitive outcomes (FSIQ). Best-fit linear models were selected as described previously, using the following variables: maternal trait anxiety at term corrected age, or mean postnatal anxiety; child's gestational age at birth; days ventilated; sex; brain MRI classification; SDQ score; SRS-2 score; child's corrected age at assessment; IMD score; and maternal education. VIF was <2 and Condition Index <2.3 for all variables in the FSIQ models. Diagnostics showed no evidence of heteroscedasticity, hence robust standard errors were not used for these analyses.

## 3 Results

Complete outcome (SDQ and/or SRS-2) and covariate data were available for 140 children and were included in the analysis. Mothers in our sample were older, and a higher proportion had attained post-16 education, compared to those not followed up in the e-Prime cohort (Table 1). The lower rate of multiple birth in our cohort (Table 2) reflects our random selection of only one of a twin/triplet set; there was no significant difference in multiple births in the 215 children followed up at 4–6 years (χ^2^ (1, *N* = 510) = 0.36, *p* = 0.55). Brain MRI classification in the follow up cohort was as follows: no lesion n=73 (51.1%); minor lesions n=53 (37.9%); major lesions n=14 (10.0%).

Children's mean FSIQ at follow-up was within the population norm (mean = 107, SD = 16) (Wechsler, 2012). Mean SDQ score was 9.8 (SD 5.9), and 32/139 children (23.0%) had an elevated (≥14) SDQ score. In comparison, the mean SDQ score in a representative British population sample of 5–10 year olds was 8.6 (SD 5.7), and 18.3% had an elevated (≥14) SDQ score (YouthinMind, 2001). Mean SRS-2 T-score was 48.6 (SD 9.0), and 13/135 children (9.6%) had elevated SRS-2 scores. The mean SRS T-score in the normative population is 50 (SD 10) (Constantino and Gruber, 2012).

Best-fit predictors of SDQ scores are shown in Table 3. Higher maternal trait anxiety at term corrected age was associated with children's higher SDQ scores, with a small-medium effect size. More advanced gestational age at birth predicted higher SDQ scores when modelled with maternal trait anxiety at term corrected age. Higher FSIQ had a significant protective effect on SDQ scores, as did higher maternal educational level. Mean postnatal maternal trait anxiety was also associated with children's higher SDQ scores, with a medium effect size (eTable 2 in the Supplement).

**Table 3:** SDQ predictors using best model with maternal trait anxiety at term corrected age.

Factor	t	β [95% CI]	P	sr^2^	f^2^
Maternal anxiety	3.01	0.25 [0.09, 0.41]	0.003 **	0.06	0.08
Gestation	2.68	0.21 [0.06, 0.36]	0.008 **	0.04	0.06
FSIQ	−4.06	−0.30 [−0.44, −0.15]	0.000 ***	0.07	0.10
Corrected age	1.89	0.19 [−0.01, 0.39]	0.06	–	–
Maternal education: higher	−2.65	−0.24 [−0.42, −0.06]	0.009 **	0.05	0.07

*p* < 0.05 *; *p* < 0.01 **; *p* < 0.001 ***.

df = 133. Model r^2^ = 0.277.

SDQ = Strengths & Difficulties Questionnaire. Outcome variable = SDQ score. Maternal anxiety = maternal trait anxiety score at term corrected age. Gestation = gestational age at birth. FSIQ = full-scale composite IQ score at pre-school age. Corrected age = age of child at assessment, corrected for gestation at birth. Maternal education: higher = left full-time education aged ≥19 years (dummy).

β = standardised coefficient.

Effect size (Cohen's f^2^, calculated from squared part correlations for predictors significant to 0.05): 0.02 = small, 0.15 = medium and 0.35 = large (Cohen, 1988).

- indicates data not given, as predictor not significant to 0.05.

Best-fit predictors of SRS-2 scores are shown in Table 4. Higher maternal trait anxiety at term corrected age was associated with children's higher SRS-2 scores, with a small effect size. Higher FSIQ had a significant protective effect on SRS-2 scores, as did higher maternal educational level. Mean postnatal maternal trait anxiety was associated with children's higher SRS-2 score, with a small effect size (eTable 3 in the Supplement).

**Table 4:** SRS-2 predictors using best model with maternal trait anxiety at term corrected age.

Factor	t	β [95% CI]	p	sr^2^	f^2^
Maternal anxiety	2.23	0.15 [0.02, 0.28]	0.03 *	0.03	0.04
Gestation	1.33	0.09 [−0.04, 0.23]	0.18	–	–
FSIQ	−5.27	−0.37 [−0.51, −0.23]	0.000 ***	0.15	0.22
Corrected age	1.40	0.15 [−0.06, 0.37]	0.16	–	–
Maternal education: higher	−2.89	−0.25 [−0.42, −0.08]	0.005 **	0.07	0.10

*p* < 0.05 *; *p* < 0.01 **; *p* < 0.001 ***.

df = 129. Model r^2^ = 0.337.

SRS-2 = Social Responsiveness Scale 2. Outcome variable = SRS-2 score. Maternal anxiety = maternal trait anxiety score at term corrected age. Gestation = gestational age at birth. FSIQ = full-scale composite IQ score at pre-school age. Corrected age = age of child at assessment, corrected for gestation at birth. Maternal education: higher = left full-time education aged ≥19 years (dummy).

β = standardised coefficient.

Effect size (Cohen's f^2^, calculated from squared part correlations for predictors significant to 0.05): 0.02 = small, 0.15 = medium and 0.35 = large (Cohen, 1988).

- indicates data not given, as predictor not significant to 0.05.

Maternal trait anxiety at term corrected age and mean postnatal maternal trait anxiety did not predict children's FSIQ (eTable 4 and eTable 5 in the Supplement, respectively).

## 4 Discussion

### 4.1 Main findings

The aim of this study was to examine the role of postnatal maternal anxiety in the development of mental health problems in children born preterm, whilst controlling for clinical and socio-demographic risk factors. This is, to our knowledge, the first longitudinal study to investigate the association between maternal anxiety and childhood behavioural problems in a cohort of preterm infants born <33 weeks, and adds to the increasing body of literature that recognises the possible role of maternal anxiety in the aetiology of neurodevelopmental disorders. In addition, this study facilitates comparison of the clinical relevance of outcome predictors using effect sizes, and provides unique insights into the role of early versus ongoing postnatal maternal anxiety in a high-risk cohort.

In line with our hypothesis, postnatal maternal trait anxiety was associated with poorer mental health outcomes in ex-preterm children at pre-school age, with respect to propensity for general psychopathology and, to a lesser extent, autism symptomatology. While the effect size of early postnatal maternal anxiety on children's general psychopathology was small-medium, higher maternal anxiety during the subsequent two years had a moderate effect, suggesting that ongoing maternal anxiety during the first years of life could impact children's behavioural and emotional development. In contrast, the effect of maternal anxiety in the early postnatal period, and thereafter, on autism symptomatology was small, possibly indicating a less robust role of postnatal anxiety in the aetiology of social communication disorders.

Our findings with respect to children's general psychopathology are consistent with those of recent studies, that maternal anxiety at 8 weeks (Polte et al., 2019), parental distress at 9 months (Hadfield et al., 2017), and maternal anxiety at 21 months postnatal (Barker et al., 2011) are associated with increased risk of social-emotional problems in offspring. High maternal anxiety during toddlerhood was also found to predict less social competency – a composite of emotional, behavioural and social-interaction measures – in pre-school-aged children who were born very preterm (Jones et al., 2013).

Although mediating mechanisms were not directly tested in our study, others have examined hypothetical behavioural and biological pathways, which may aid the understanding of our – and others’ – results linking postnatal maternal anxiety to childhood general psychopathology. Behaviourally, maternal anxiety has been associated with poorer maternal-infant bonding (Tietz et al., 2014), fewer positive and more negative maternal interactions (Stevenson-Hinde et al., 2013), and less maternal sensitivity (Kertz et al., 2008; Leerkes et al., 2009; Nicol-Harper et al., 2007; Stevenson-Hinde et al., 2013). Maternal sensitivity, in turn, may mediate the effect of maternal anxiety on security of attachment (Stevenson-Hinde et al., 2013). Insecure attachment has been linked to the development of childhood behavioural problems (Fearon et al., 2010), possibly via aberrant social and emotional regulation (Boldt et al., 2020; Cooke et al., 2019), and may thus underlie our observed association between maternal anxiety and childhood behavioural outcomes. Biological changes have also been recognised in children of mothers who experienced perinatal stress and anxiety, including elevated cortisol reactivity (Feldman et al., 2009; Simons et al., 2019), and microstructural white matter alterations in brain regions involved in cognitive-emotional responses to stress and sensory processing, as well as socio-emotional function (Lautarescu et al., 2020; Rifkin-Graboi et al., 2015). These microstructural alterations have been observed at term-corrected age in the same cohort of preterm infants used for our study, although stressful life events, not trait anxiety, were related to the changes (Lautarescu et al., 2020). Of course, psychiatric (Biel, 2018; Tietz et al., 2014), social (Ronfani et al., 2015), genetic (Assary et al., 2018; Hallmayer et al., 2011), and perinatal (Belfort et al., 2016; Sotiriadis et al., 2015) factors may confound these observed associations, and further research is needed to fully understand the interplay of biochemical and neurostructural changes in the translation of environmental exposures into distal behavioural phenotypes.

Our finding of a small effect size of postnatal maternal anxiety on autism symptomatology in children at pre-school age is reflected by the lack of substantial literature in this field. ASD has been linked to various parental psychopathologies including neurotic disorders (Jokiranta et al., 2013), and there are suggestions that postnatal depression and anxiety are pertinent factors in cross-domain studies of ASD (Babb et al., 2015). Self-reported antenatal maternal anxiety and depression have also been associated with the dysregulated phenotype of childhood autism (Wiggins et al., 2019), suggesting that maternal mental state could influence the symptom profile exhibited by children with ASD. However, these studies do not highlight a clear aetiological role of postnatal maternal anxiety in the development of autism *per se*.

Maternal trait anxiety was selectively associated with children's mental health outcomes, and not with FSIQ. While this contrasts with existing work (Tarabulsy et al., 2014), possibly due to the authors' focus on cognitive development rather than IQ, our other predictors of lower FSIQ – higher SRS-2 score, more days ventilated on NICU, and greater socio-economic deprivation – are reflective of the current literature (Beauregard et al., 2018; Hus et al., 2013; Twilhaar et al., 2018). The relevance of IQ and maternal education in our models for predicting childhood mental health problems also reflects the existing literature, that higher IQ and maternal education are associated with better outcomes (Davis et al., 2010; Flouri et al., 2018; Plomin et al., 2002; Sonego et al., 2013); the large effect sizes of IQ on SRS-2 scores are particularly prominent.

Gestational age at birth also predicted SDQ score in pre-school children. Firstly of note, although its effect size was relatively small, the direction of the model regression coefficients indicates that longer gestation, within this preterm cohort, may predict a worse behavioural outcome. In contrast, a recent meta-analysis showed both moderately and very prematurely-born infants having similarly increased risk of developing ADHD, when compared to term babies (Allotey et al., 2018). It is possible that this discrepancy may be due to issues associated with statistical power, as our sample size is considerably smaller than those of other studies, or due to model specification. Our model outcome was a non-diagnostic measure of general psychopathology; therefore, it may not be directly comparable to findings referring to an ADHD diagnosis (e.g., Allotey et al., 2018). The second point of interest is that gestational age at birth did not predict autism symptomatology, and the effect sizes of gestation on SDQ scores are smaller than those of maternal anxiety in the early and ongoing postnatal period. This suggests that postnatal maternal anxiety may be of greater clinical significance in determining childhood mental health outcomes in preterm children than the degree of prematurity. Previous research also suggests that gestational age, unlike postnatal parental emotional distress, is not a significant predictor of childhood socioemotional outcomes (Hadfield et al., 2017). These findings, if further corroborated, support the need to improve long-term risk-stratification for preterm infants, incorporating measures of postnatal maternal mental health, to identify and support families with infants at particular risk of neurodevelopmental and mental health disorders.

### 4.2 Strengths & limitations

The strengths of this study rest on its unique cohort of very high-risk infants, together with the wealth of information on confounding factors that was available for the analysis. Given the highly complex, multi-factorial aetiology of neurodevelopmental disorders, particularly in premature children, such a dataset is incredibly valuable. Moreover, using a best-model fit regression analysis provides an understanding of the variables that best explain the relationship observed with behavioural outcomes, and effect sizes allow comparison between significant model predictors.

However, it is likely that the effect of postnatal maternal anxiety on child neurodevelopment is more complex. Multiple confounders that were not included in this study are likely to affect a child's behavioural outcome, such as antenatal anxiety (Polte et al., 2019; Tarabulsy et al., 2014), shared genetic risk for mental illness (The Brainstorm Consortium, 2018), mother-infant interaction (Nicol-Harper et al., 2007), attachment (Stevenson-Hinde et al., 2013), comorbid maternal depression (Tietz et al., 2014), paternal mental health (Kvalevaag et al., 2013; Ramchandani et al., 2008), family structure, parental employment (Davis et al., 2010), child abuse and neglect (Hunt et al., 2017). In addition, we were unable to include early and ongoing postnatal maternal anxiety in the same regression model due to multicollinearity, and are therefore unable to firmly conclude whether early or later maternal anxiety has the greatest impact on childhood mental health outcomes.

This study did not have a control group of term infants, and thus cannot draw inference about the impact that postnatal maternal anxiety has on preterm versus term infants and their respective mental health outcomes. It also limits the generalisability of conclusions about the degree of prematurity to a specific population of preterm newborns.

The outcome measures of this study were derived from parent-completed questionnaires and it is possible that our results are skewed by reporting bias, as poorer maternal mental health has been associated with inflated reporting of behavioural problems in children (Najman et al., 2001). Whilst Harman's single factor test indicated that common method bias did not affect our data, further studies could include home- or lab-based observations, to provide objective assessments of child behaviour. In addition, it must be noted that we assessed maternal tendency to react anxiously and not clinical anxiety. In fact, there is debate over whether the STAI-trait reflects general negative affect more than anxiety (Bados et al., 2010). Selection bias likely arose at point of recruitment into the e-Prime study (Edwards et al., 2018), as well as through subsequent participant drop-out. Our sample of 140 infants analysed for this study also differed slightly from the initial e-Prime cohort with respect to socio-demographic characteristics, which limits the representativeness of the sample and may restrict generalisability of results to the wider population of preterm neonates. Finally, the number of children with elevated scores – particularly SRS-2 scores – was limited, and the effect size of maternal trait anxiety on outcomes relatively small; the clinical significance of our findings will require further investigation.

## 5 Conclusion

Our results support the growing body of evidence highlighting the role of postnatal maternal mental distress in determining children's mental health outcomes. Importantly, we describe this association in a population of high-risk infants, which has implications for clinical care in NICU and at infant follow-up. Clinicians have a valuable window of opportunity to mitigate any avoidable detrimental effect on children's development, by optimising and personalising neonatal and follow-up care to target modifiable aetiological factors.

Further research is needed to substantiate the effect of maternal postnatal anxiety on child behavioural outcomes in preterm babies, controlling for extensive ante- and postnatal variables. The robustness of our observed small-medium effects also requires confirmation, in order to determine the true neurodevelopmental and clinical implications of postnatal maternal anxiety. Simultaneously, paternal mental state is critical to investigate in the pursuit of understanding the balance of multiple – inherited, acquired and environmental – factors in child neurodevelopment. Exploring interventions that best mitigate the risk of developmental psychopathology will require further work to assess possible protective factors, as well as developing effective multi-disciplinary team approaches that provide holistic, preventative care to neonates, children and their families.

## Contributors

**Ira Kleine**: Formal analysis, Writing – Original draft, Visualisation.

**Shona Falconer**: Investigation, Writing – Review & Editing.

**Simon Roth**: Investigation, Writing – Review & Editing.

**Serena Counsell**: Conceptualization, Methodology, Formal analysis, Writing – Review & Editing, Funding acquisition.

**Maggie Redshaw**: Investigation, Writing – Review & Editing.

**Nigel Kennea**: Investigation, Writing – Review & Editing.

**Anthony David Edwards**: Conceptualization, Methodology, Writing – Review & Editing, Supervision, Funding acquisition.

**Chiara Nosarti**: Conceptualization, Methodology, Formal analysis, Writing – Reviewing & Editing, Supervision.

## Declaration of competing interest

None.
