# Supplementary material for: Early postnatal maternal trait anxiety is associated with the behavioural outcomes of children born preterm <33 weeks
Source: J Psychiatr Res. 2020 Dec;131:160–8. doi: 10.1016/j.jpsychires.2020.09.010 (PMC7676467; doi:10.1016/j.jpsychires.2020.09.010)
Supplement: Multimedia component 1 [file mmc1.docx]

**Supplementary material**

**eTable 1. Two-tailed Pearson correlation coefficients for continuous variables of interest.**

**eTable 2. SDQ predictors using best model with mean postnatal maternal trait anxiety.**

**eTable 3. SRS-2 predictors using best model with mean postnatal maternal trait anxiety.**

**eTable 4. FSIQ predictors using best model with maternal trait anxiety at term corrected age.**

**eTable 5. FSIQ predictors using best model with mean postnatal maternal trait anxiety.**

**eTable 1. Two-tailed Pearson correlation coefficients for continuous variables of interest.**

|  | **1** | **2** | **3** | **4** | **5** | **6** | **7** | **8** | **9** | **10** | **11** | **12** | **13** | **14** |
| --- | --- | --- | --- | --- | --- | --- | --- | --- | --- | --- | --- | --- | --- | --- |
| **1. Maternal trait term** | 1 |  |  |  |  |  |  |  |  |  |  |  |  |  |
| **2. Maternal trait 14d** | 0.78** | 1 |  |  |  |  |  |  |  |  |  |  |  |  |
| **3. Maternal trait 12m** | 0.64** | 0.69** | 1 |  |  |  |  |  |  |  |  |  |  |  |
| **4. Maternal trait 22m** | 0.63** | 0.66** | 0.74** | 1 |  |  |  |  |  |  |  |  |  |  |
| **5. Maternal trait**  **mean postnatal** | 0.75** | - | - | - | 1 |  |  |  |  |  |  |  |  |  |
| **6. Gestation** | -0.12 | -0.05 | 0.04 | 0.01 | -0.01 | 1 |  |  |  |  |  |  |  |  |
| **7. FSIQ** | 0.10 | 0.01 | -0.02 | 0.08 | 0.02 | 0.09 | 1 |  |  |  |  |  |  |  |
| **8. Corrected age** | 0.07 | 0.10 | 0.08 | 0.07 | 0.08 | -0.19* | -0.20* | 1 |  |  |  |  |  |  |
| **9. IMD score** | -0.09 | -0.10 | -0.11 | -0.15 | -0.13 | -0.02 | -0.23** | 0.00 | 1 |  |  |  |  |  |
| **10. Days ventilated** | -0.00 | -0.01 | -0.05 | -0.01 | -0.02 | -0.58** | -0.18* | 0.16 | 0.05 | 1 |  |  |  |  |
| **11. Days TPN** | 0.08 | 0.02 | -0.04 | 0.01 | 0.01 | -0.63** | -0.12 | 0.25** | 0.03 | 0.78** | 1 |  |  |  |
| **12. Birthweight** | -0.09 | -0.06 | -0.10 | -0.05 | -0.07 | 0.80** | 0.08 | -0.17* | 0.07 | -0.44** | -0.53** | 1 |  |  |
| **13. SDQ score** | 0.21* | 0.26** | 0.37** | 0.24** | 0.30** | 0.12 | -0.33** | 0.22** | 0.08 | 0.00 | -0.04 | 0.05 | 1 |  |
| **14. SRS-2 score** | 0.14 | 0.22* | 0.22* | 0.06 | 0.18* | -0.01 | -0.46** | 0.24** | 0.16 | 0.06 | -0.04 | 0.02 | 0.65** | 1 |

1 – Maternal trait anxiety at term corrected age; 2 – Maternal trait anxiety at 14 days; 3 – Maternal trait anxiety at 12 months; 4 – Maternal trait anxiety at 22 months; 5 – Maternal trait anxiety mean postnatal; 6 – Gestational age at birth; 7 – Full-scale IQ score; 8 – Age at assessment, corrected for gestation at birth; 9 – Index of Multiple Deprivation (IMD) score; 10 – Days ventilated; 11 – Days given Total Parenteral Nutrition (TPN); 12 – Birthweight; 13 – Strengths and Difficulties Questionnaire (SDQ) total score; 14 – Social Responsiveness Scale 2 (SRS-2) total score.

p<0.05 * ; p<0.01 **

**eTable 2. SDQ predictors using best model with mean postnatal maternal trait anxiety.**

| **Factor** | **t** | **ß**  **[95% CI]** | **p** | **sr^2^** | **f^2^** |
| --- | --- | --- | --- | --- | --- |
| Maternal anxiety (mean) | 3.46 | 0.30  [0.13, 0.47] | 0.001  *** | 0.09 | 0.13 |
| Gestation | 2.39 | 0.18  [0.03, 0.33] | 0.02  * | 0.03 | 0.05 |
| FSIQ | -3.73 | -0.28  [-0.42, -0.13] | 0.000  *** | 0.06 | 0.09 |
| Corrected age | 1.67 | 0.18  [-0.03, 0.40] | 0.10 | - | - |
| Maternal education: higher | -2.81 | -0.25  [-0.43, -0.08] | 0.006  ** | 0.05 | 0.08 |

p<0.05 *; p<0.01 **; p<0.001 ***.

Data for mean postnatal maternal STAI and child SDQ score was available for 139 participants.

df = 133. Model r^2^ = 0.305

SDQ = Strengths & Difficulties Questionnaire. Outcome variable = SDQ score. Maternal anxiety (mean) = mean postnatal maternal trait anxiety score, computed from available trait anxiety scores at 14 days, 12 months and 22 months corrected age. Gestation = gestational age at birth. FSIQ = full-scale composite IQ score at pre-school age. Corrected age = age of child at assessment (years), corrected for gestation at birth. Maternal education: higher = left full-time education aged ≥19 years (dummy).

ß = standardised coefficient.

Effect size (Cohen’s f^2^, calculated from squared part correlations for predictors significant to 0.05): 0.02 = small, 0.15 = medium and 0.35 = large. (Cohen, 1988)

- indicates data not given, as predictor not significant to 0.05.

**eTable 3. SRS-2 predictors using best model with mean postnatal maternal trait anxiety.**

| **Factor** | **t** | **ß**  **[95% CI]** | **p** | **sr^2^** | **f^2^** |
| --- | --- | --- | --- | --- | --- |
| Maternal anxiety (mean) | 2.06 | 0.14  [0.01, 0.27] | 0.04  * | 0.02 | 0.04 |
| FSIQ | -4.89 | -0.35  [-0.49, -0.21] | 0.000  *** | 0.13 | 0.19 |
| Corrected age | 1.25 | 0.14  [-0.08, 0.36] | 0.21 | - | - |
| Maternal education: higher | -2.95 | -0.25  [-0.41, -0.08] | 0.004  ** | 0.07 | 0.10 |

## p<0.05 *; p<0.01 **; p<0.001 ***.

Data for mean postnatal maternal STAI and child SRS-2 score was available for 135 participants.

df = 130. Model r^2^ = 0.326

SRS-2 = Social Responsiveness Scale 2. Outcome variable = SRS-2 score. Maternal anxiety (mean) = mean postnatal maternal trait anxiety score, computed from available trait anxiety scores at 14 days, 12 months and 22 months corrected age. FSIQ = full-scale composite IQ score at pre-school age. Corrected age = age of child at assessment, corrected for gestation at birth. Maternal education: higher = left full-time education aged ≥19 years (dummy).

ß = standardised coefficient.

Effect size (Cohen’s f^2^, calculated from squared part correlations for predictors significant to 0.05): 0.02 = small, 0.15 = medium and 0.35 = large. (Cohen, 1988)

- indicates data not given, as predictor not significant to 0.05.

**eTable 4. FSIQ predictors using best model with maternal trait anxiety at term corrected age.**

| **Factor** | **t** | **ß**  **[95% CI]** | **p** | **sr^2^** | **f^2^** |
| --- | --- | --- | --- | --- | --- |
| Maternal anxiety (term) | 1.75 | 0.13  [-0.02, 0.27] | 0.08 | - | - |
| SRS-2 | -3.62 | -0.39  [-0.60, -0.18] | 0.000  *** | 0.07 | 0.10 |
| SDQ | -1.00 | -0.09  [-0.28, 0.09] | 0.32 | - | - |
| IMD score | -2.20 | -0.16  [-0.30, -0.02] | 0.03  * | 0.03 | 0.04 |
| Days ventilated | -2.59 | -0.27  [-0.47, -0.06] | 0.01  * | 0.04 | 0.05 |
| Minor MRI lesions | 1.72 | 0.12  [-0.02, 0.26] | 0.09 | - | - |

p<0.05 *; p<0.01 **; p<0.001 ***.

Data for maternal STAI at term corrected age, FSIQ, SDQ and SRS-2 score was available for 134 participants.

df = 127. Model r^2^ = 0.302.

FSIQ = full-scale composite IQ score at pre-school age. Outcome variable = FSIQ.

Maternal anxiety (term) = maternal trait anxiety score at term corrected age. SRS-2 = Social Responsiveness Scale 2 score. SDQ = Strengths & Difficulties Questionnaire score. IMD score = Index of Multiple Deprivation (IMD) score. Days ventilated = total days ventilated whilst on NICU. Minor MRI lesions = minor brain lesions identified on MRI scan at term-corrected age.

ß = standardised coefficient.

Effect size (Cohen’s f^2^, calculated from squared part correlations for predictors significant to 0.05): 0.02 = small, 0.15 = medium and 0.35 = large. (Cohen, 1988)

- indicates data not given, as predictor not significant to 0.05.

**eTable 5. FSIQ predictors using best model with mean postnatal maternal trait anxiety.**

| **Factor** | **t** | **ß**  **[95% CI]** | **p** | **sr^2^** | **f^2^** |
| --- | --- | --- | --- | --- | --- |
| SRS-2 | -3.57 | -0.39  [-0.60, -0.17] | 0.001  *** | 0.07 | 0.10 |
| SDQ | -0.71 | -0.07  [-0.25, 0.12] | 0.48 | - | - |
| IMD score | -2.35 | -0.17  [-0.31, -0.03] | 0.02  * | 0.03 | 0.04 |
| Days ventilated | -2.58 | -0.27  [-0.47, -0.06] | 0.01  * | 0.04 | 0.05 |
| Minor MRI lesions | 1.72 | 0.12  [-0.02, 0.26] | 0.09 | - | - |

p<0.05 *; p<0.01 **; p<0.001 ***.

Data for mean postnatal maternal STAI, FSIQ, SDQ and SRS-2 score was available for 134 participants.

df = 128. Model r^2^ = 0.286.

FSIQ = full-scale composite IQ score at pre-school age. Outcome variable = FSIQ.

SRS-2 = Social Responsiveness Scale 2 score. SDQ = Strengths & Difficulties Questionnaire score. IMD score = Index of Multiple Deprivation (IMD) score. Days ventilated = total days ventilated whilst on NICU. Minor MRI lesions = minor brain lesions identified on MRI scan at term-corrected age.

ß = standardised coefficient.

Effect size (Cohen’s f^2^, calculated from squared part correlations for predictors significant to 0.05): 0.02 = small, 0.15 = medium and 0.35 = large. (Cohen, 1988)

- indicates data not given, as predictor not significant to 0.05.

**Supplementary material references**

Cohen, J., 1988. Statistical power analysis for the behavioral sciences, 2nd ed. ed. L. Erlbaum Associates, Hillsdale, N.J.
